# Computational pulmonary edema: A microvascular model of alveolar capillary and interstitial flow

**DOI:** 10.1063/5.0158324

**Published:** 2023-07-05

**Authors:** James B. Grotberg, Francesco Romanò

**Affiliations:** 1 Department of Biomedical Engineering, University of Michigan, Ann Arbor, Michigan 48109, USA; 2 Université Lille, CNRS, ONERA, Arts et Métiers Institute of Technology, Centrale Lille, UMR 9014 LMFL-Laboratoire de Mécanique des Fluides de Lille-Kampé de Fériet, F-59000 Lille, France

## Abstract

We present a microvascular model of fluid transport in the alveolar septa related to pulmonary edema. It consists of a two-dimensional capillary sheet coursing by several alveoli. The alveolar epithelial membrane runs parallel to the capillary endothelial membrane with an interstitial layer in between, making one long septal tract. A coupled system of equations uses lubrication theory for the capillary blood, Darcy flow for the porous media of the interstitium, a passive alveolus, and the Starling equation at both membranes. Case examples include normal physiology, cardiogenic pulmonary edema, acute respiratory distress syndrome (ARDS), hypoalbuminemia, and effects of PEEP. COVID-19 has dramatically increased ARDS in the world population, raising the urgency for such a model to create an analytical framework. Under normal conditions fluid exits the alveolus, crosses the interstitium, and enters the capillary. For edema, this crossflow is reversed with fluid leaving the capillary and entering the alveolus. Because both the interstitial and capillary pressures decrease downstream, the reversal can occur within a single septal tract, with edema upstream and clearance downstream. Clinically useful solution forms are provided allowing calculation of interstitial fluid pressure, crossflows, and critical capillary pressures. Overall, the interstitial pressures are found to be significantly more positive than values used in the traditional physiological literature. That creates steep gradients near the upstream and downstream end outlets, driving significant flows toward the distant lymphatics. This new physiological flow provides an explanation to the puzzle, noted since 1896, of how pulmonary lymphatics can function so far from the alveoli: the interstitium is self-clearing.

## INTRODUCTION

**FIG. 1.**
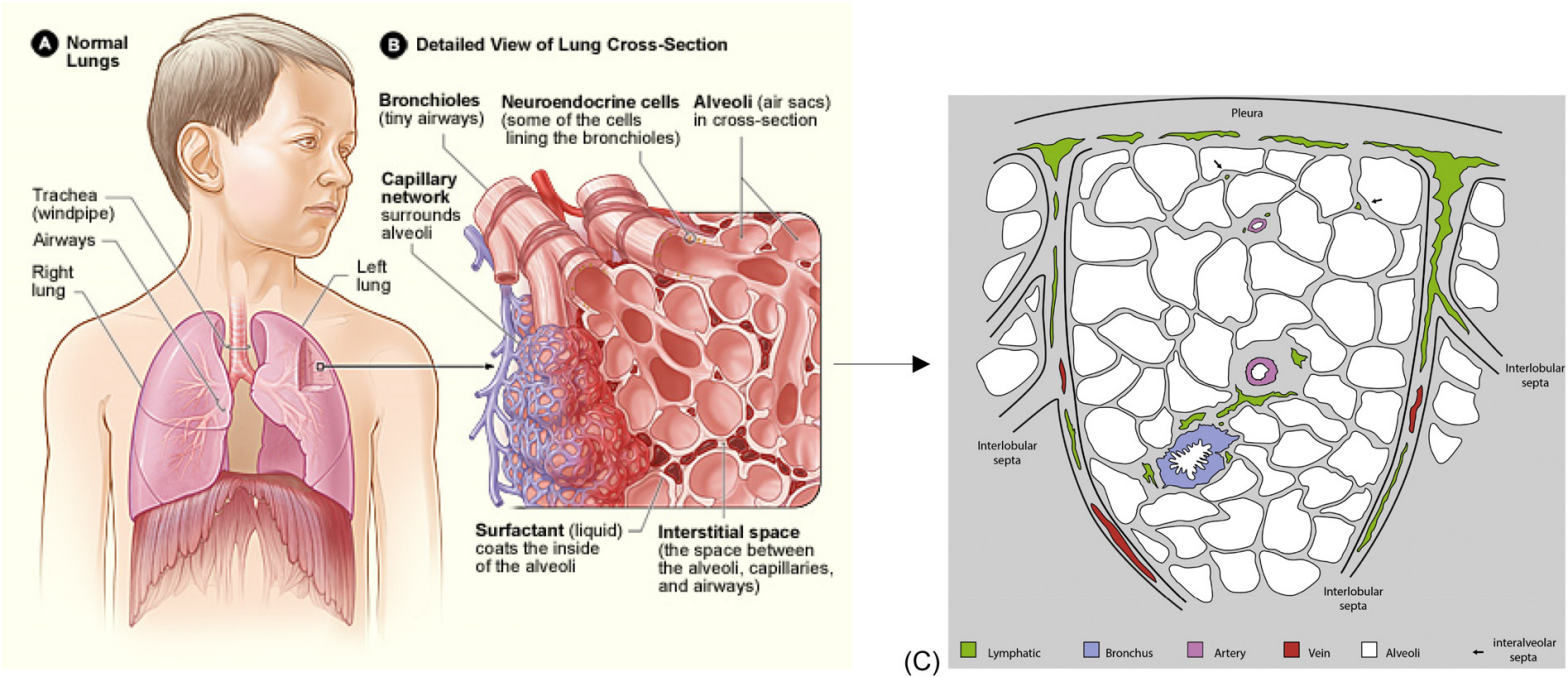
Lung anatomy: (a) the location of the lungs and airways in the body and (b) a detailed view of the lung structures such as the bronchioles, neuroendocrine cells, alveoli, capillary network, alveolar liquid with surfactant, and interstitial space. Wikimedia Commons, see https://commons.wikimedia.org/wiki/File:Lung_structure_normal.jpg for “Lung Structure Normal.jpg” (last accessed November 12, 2013).^1^ (c) Pulmonary lobular anatomy showing the alveoli, interalveolar septa, arteries, veins, bronchi, interlobular septa, lymphatics, and pleura. The distribution of lymphatic vessels (green) in the pulmonary lobule. Most vessels are located in the pleura, in the interlobular septa, and in association with bronchovascular bundles. Lymphatic vessels are also present in interalveolar septa, in association with arterioles and only occasionally independent of blood vessels. Arrows indicate lymphatic vessels independent of blood vessels in interalveolar septa. Reprinted with permission from Weber *et al.*, Ann. Anat. 218, 110–117 (2018). Copyright 2018 Elsevier.

Lungs provide the interface between inhaled air and circulating blood for the exchange of oxygen and carbon dioxide. Figure 1(a) shows the gross lung anatomy in the chest including the left and right lung and parts of the airway tree (trachea, large airways), while Fig. 1(b) is a small scale view including bronchioles, neuroendocrine cells, alveoli, capillary network, alveolar liquid with surfactant, and interstitial space. The bronchioles deliver air into and out of the alveolar sacs with ventilation. The capillary network runs adjacent to the alveoli within the interalveolar septa where the interstitium also resides. Gas exchange occurs across the septa between the blood and air compartments, and, overall, deoxygenated (blue) blood in the pulmonary arteries is returned as oxygenated (red) blood in the pulmonary veins.

**FIG. 2.**
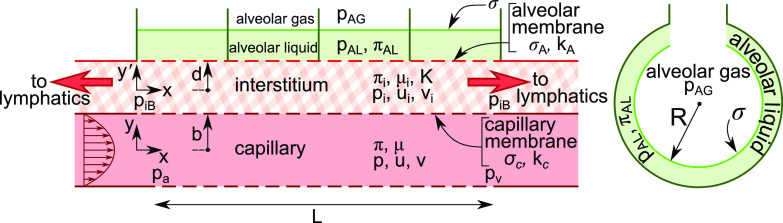
A two-dimensional model of a septal tract with capillary, interstitium, and alveolar compartments (left). Red arrows indicate flow to the lymphatics. Example of a spherical alveolus (right).

Figure 1(c) and Ref. 2 is a sketch of the pulmonary lobular anatomy in cross section. The lobule is fed by a terminal bronchiole that branches into respiratory bronchioles. The alveoli (white) are on the order of 100 μm in diameter, and the interalveolar septa (gray) are 12±3 μm thick.^3,4^ The capillary network runs within the septa and passes by several alveoli, see Fig. 1. Based on a review,^5^ the capillary path length ranges from 250−850 μm in several mammalian species.^6–8^ We call this capillary path with the adjacent septal structures the “septal tract,” see Fig. 2. The lymphatics (green) in Fig. 1(c) are generally hundreds of micrometers away from the furthest alveolar septa, significantly farther than what is found in systemic tissues where the blood capillaries and the lymphatic capillaries are within tens of micrometers from one another. Systemic interstitial fluid velocities have been measured in the systemic circulation in the range 0.1–0.5 μm/s for intradermal mouse tumors^9^ and 0–2 μm/s, with an average of 0.6±0.2  μm/s in a rabbit ear preparation.^10^ The shear stress induced by the systemic filtration flow can be in the range 0.005–0.015 dyn/cm^2.^^11^

Plasma fluid, which exits from the capillaries into the interstitium, is generally cleared by the lymphatic system. How that actually happens has been a puzzle since 1896 because of the long distances.^12^ The present model addresses this fundamental issue of lung physiology. When clearance is overwhelmed, fluid can enter the alveolar air space causing pulmonary edema, resulting in breathing difficulty that can be fatal. In today's global pandemic environment, COVID-19 is a devastating source of this failure. In this study, we present a microvascular, fluid mechanical model for this flow and explore normal and pathological situations. As part of the mathematical process, we calculate the interstitial fluid pressure, $p_{i}$, which has never been directly measured or previously calculated. We find it is quite different from traditional values that are commonly used by physiologists and physicians. An additional feature is the discovery of comparatively large interstitial fluid velocities exiting the ends of the septal tract, a newly identified mechanism for enhancing clearance.

The interstitium, as a component of the barrier, is also found throughout the body where it separates capillaries from tissue epithelium. Known alternatively as the extracellular matrix (ECM) the interstitium can be treated fluid mechanically as a simple porous media^13–15^ or a more complex viscoelastic porous media.^16^ The solid component of the interstitium consists of tangled fibrils primarily of collagen, but also elastin and glycosaminoglycans, which are cross-linked to form a supportive structure. Overproduction of collagen in the lung setting can lead to idiopathic pulmonary fibrosis (IPF).^17–20^ As the name suggests, the origin of IPF, an insidious fatal disease with no cure, is not known. However, flow over fibroblasts can affect their function,^21,22^ which has not been examined in the lung. The fluid component of the interstitium is similar to plasma.

Throughout the body, blood capillaries are normally permeable. They allow plasma to exit or re-enter depending on the balance of hydrostatic and osmotic (oncotic) pressures relative to the surrounding interstitium. For the systemic blood circulation, typically plasma leaks into the interstitium upstream, where hydrostatic pressures are higher, and it either re-enters the capillary downstream, where hydrostatic pressures are lower, or is absorbed by lymphatic capillaries. This is called the filtration flow. It assists in transport of gases, nutrients, and metabolic waste products between the blood and tissues. Excess outflow, not returned directly to the capillary, is collected by the lymphatic capillaries which directly overlay the blood capillary bed in the systemic system. It is pumped through a series of lymph vessels with contractile elements and one-way valves, then through nodes and ducts, finally returning to the blood circulation.^23^ The diameters of the blood capillaries are ∼5 –10 μm, while those of the lymph capillaries are ∼50 μm. Because of the close proximity, the travel distance for the interstitial fluid from blood to lymph is of similar order, i.e., tens of micrometers. The pumping action creates a cyclical pressure variation at the entrance to the lymphatic capillary. Part of the cyclical pressure is negative compared to the surrounding fluid so there is flow into the lymphatics during the phase but no outflow due to one-way valves. The circuit from capillary to interstitial fluid to lymph and back to capillary has an overall flow rate estimated to be 8 l per day for the entire body. A typical blood volume for an adult, which includes red blood cells and plasma, is ∼5 l. For a normal hematocrit of 40% red blood cells, the intravascular plasma is ∼3 liters, so all of it is recycled once every ∼9 h.^24–27^ Typical values of lung lymphatic flow are 5–10 ml/h in sheep and estimated to be 10–20 ml/h in humans.^28^ This flow balances the net filtration out of the capillaries derived from the flow rate out, 100ml/h. minus the flow rate in, 80 ml/h, = 20 ml/h net out. These lymph flow rates can increase several folds in response to increased net filtration.^29,30^

**FIG. 3.**
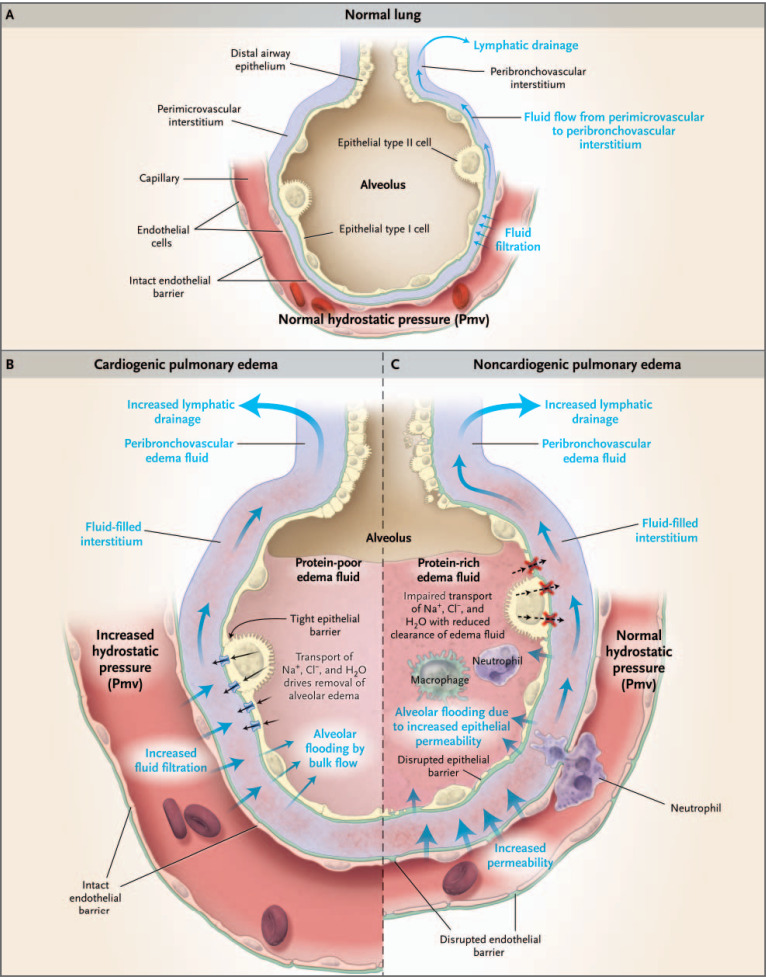
Physiology of microvascular fluid exchange in the lung. (a) Normal alveolus and capillary and pulmonary edema from (b) cardiogenic and (c) non-cardiogenic origins. Reprinted with permission from Ware *et al.*, New England J. Med. **353**, 2788–2796 (2005). Copyright 2005 Massachusetts Medical Society.

Non-cardiogenic causes of pulmonary edema are relevant to the current COVID-19 pandemic. One of the major pulmonary targets of COVID-19 are the alveolar type II cells,^31^ which, along with alveolar type I cells, form the alveolar membrane separating gas from blood, see Fig. 3(a). Because alveolar type II cells produce surfactant, patients can experience a primary surfactant deficiency and collapsed lung regions from the higher surface tensions.^32^ We will see that surfactant deficiency also plays a role in pulmonary edema formation. Surfactant replacement therapy by tracheal or bronchoscopic instillation of exogenous surfactants into the lung is a potential means of treating this deficiency.^33–38^

In addition to damaging alveolar type II cells in their role as a barrier, the general inflammatory process disrupts capillary membranes, increasing their permeability. So both boundaries, capillary endothelium and alveolar epithelium, are disrupted. The overall picture leads to acute lung injury (ALI). ALI and acute respiratory distress syndrome (ARDS), in general, are associated with sepsis, pneumonia, aspiration of gastric contents, and major trauma of the lung or non-lung structures. The resulting pulmonary edema is a protein-rich fluid which creates severe hypoxemia and bilateral infiltrates seen in chest x-rays.^39–42^ Prior to the COVID-19 pandemic, the incidence of ALI/ARDS in the US has been estimated as 200 000 cases/year, and the mortality rate varies in the range of 40%, so ∼80 000 deaths/year.^43–45^ However, cases and mortality around the world have skyrocketed into the millions since the pandemic initiated in early 2020.

The basic flow in the alveolar interstitium, having permeable capillary endothelial and alveolar epithelial boundaries, with a background lymphatic drainage is a complicated fluid mechanical environment. There are compartmental models of pulmonary interstitial and lymphatic flows utilizing resistance and compliance components,^46–48^ including effects of ventilatory motion.^49^ However, in the face of a worldwide pandemic, there is compelling motivation to establish and investigate a detailed fluid mechanical model.

## RESULTS

To explore the model, it is important to choose parameter values that are representative of common respiratory conditions, both healthy and diseased. Normal physiology, case (a), shown inFigs. 4–6, uses the values calculated in section Introduction and listed in Nomenclature. This is the base set. A sketch of the normal alveolus is shown in Fig. 3(a).^93^ Note the thin liquid lining the inside of the alveolus, which has a surfactant layer, the type I and type II epithelial cells forming the alveolar wall, and the capillary with red blood cells and its endothelial wall, and the interstitium in between. The remaining figures are for pathological conditions.

**FIG. 4.**
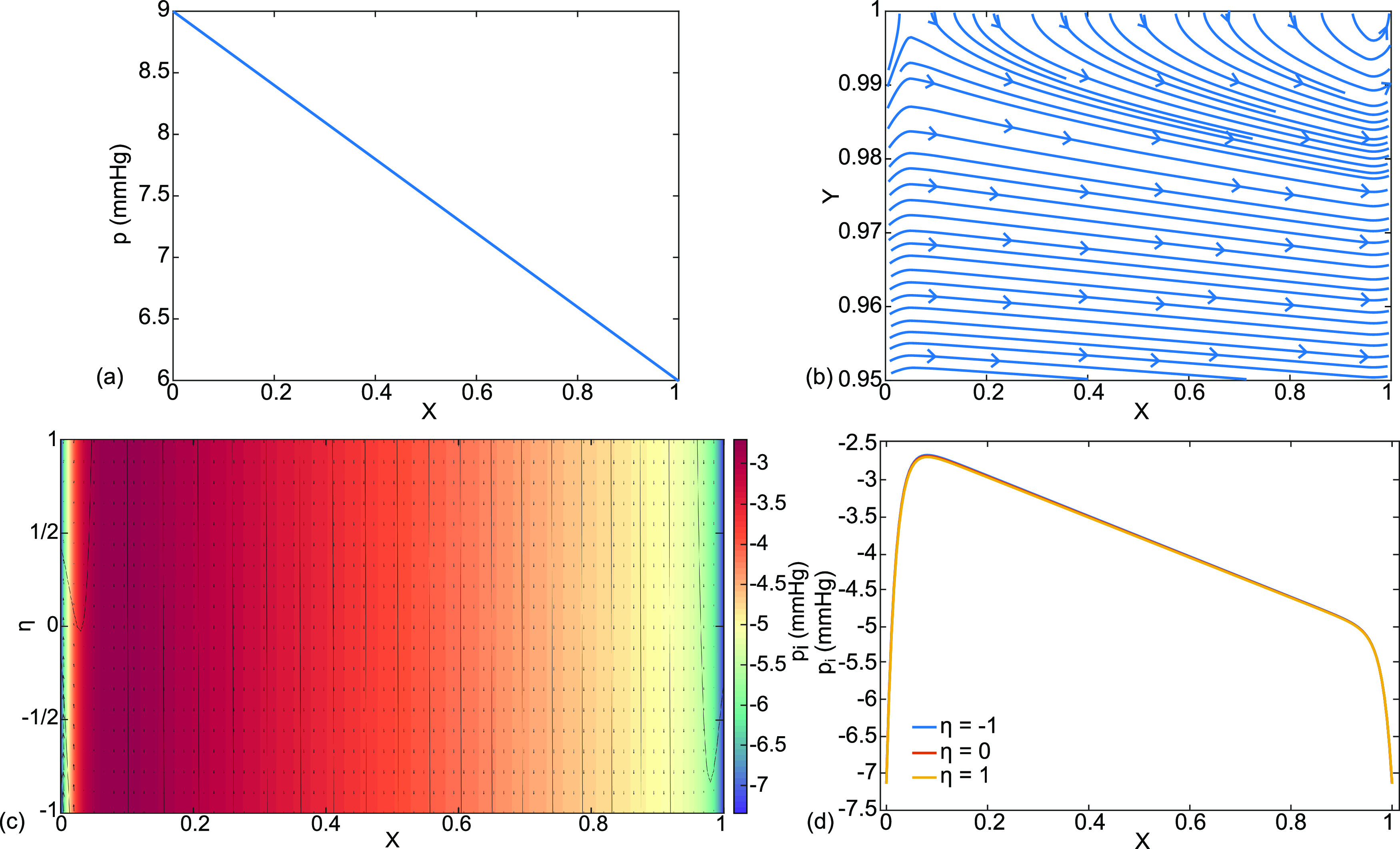
This is case (a), normal physiology, with parameter choices from Nomenclature, the base state. The results are (a) dimensional capillary pressure p(X) in mm Hg, (b) dimensionless capillary velocity vector field (U,V) and streamlines, (c) interstitial velocity vector field $\left(U_{i\;},V_{i\;}\right)$, streamlines, and pressure distribution $p_{i}$ color coded in mm Hg, and (d) interstitial fluid pressure $p_{i\;}(X,\eta =-1,0,1)$ in mm Hg.

**FIG. 5.**
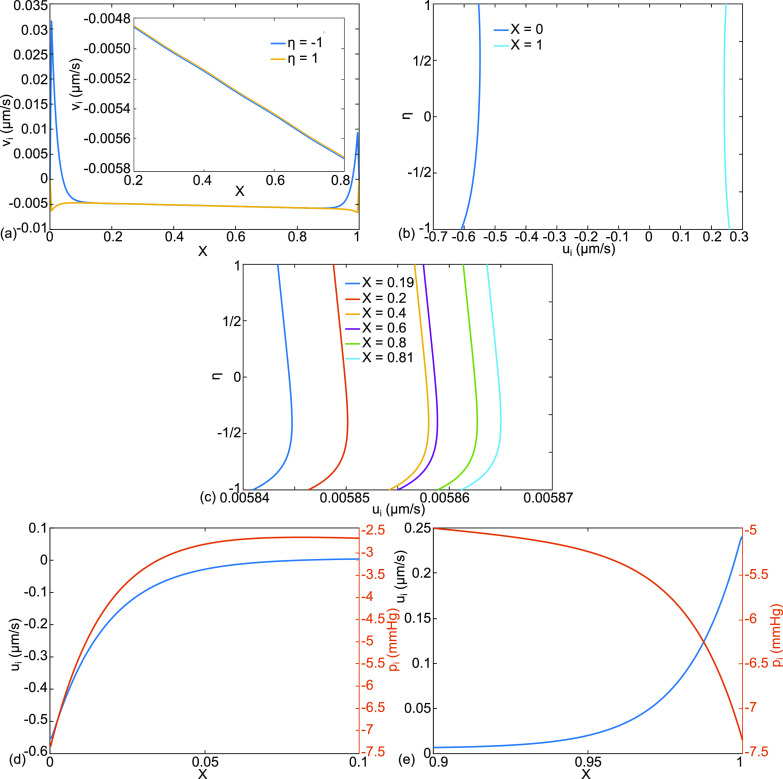
Continuing with case (a), normal physiology, the additional results are (a) alveolar membrane crossflow velocity, $v_{i\;}\left(\eta =1\right)$, and capillary membrane crossflow velocity, $v_{i\;}\left(\eta =-1\right)$ in μm/s with an inset for 0.2≤X≤0.8, (b) X-velocity $u_{i\;}\left(X,\eta \right)$ for X=0,1,(c) X-velocity $u_{i\;}\left(X,\eta \right)$ for X=0.19,0.2,0.4,0.6,0.8,0.81, (d) $u_{i\;}(X,\eta =0)\;,\;p_{i\;}(X,\eta =0)$ for 0≤X≤0.1, and (e) $u_{i\;}(X,\eta =0)\;,p_{i\;}(X,\eta =0)$ for 0.9≤X≤1.

**FIG. 6.**
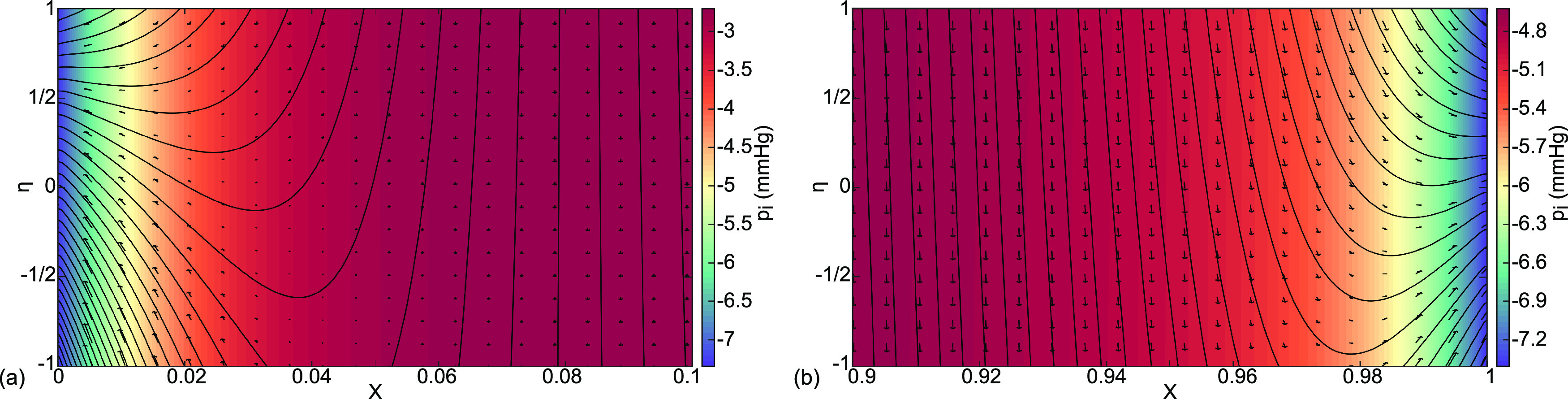
Case (a) interstitial velocity vector field $\left(U_{i\;},V_{i\;}\right)$, streamlines, and pressure distribution $p_{i}$ color coded in mm Hg for (a) the boundary layer at 0≤X≤0.1 and (b) the boundary layer at 0.9≤X≤1.

**FIG. 7.**
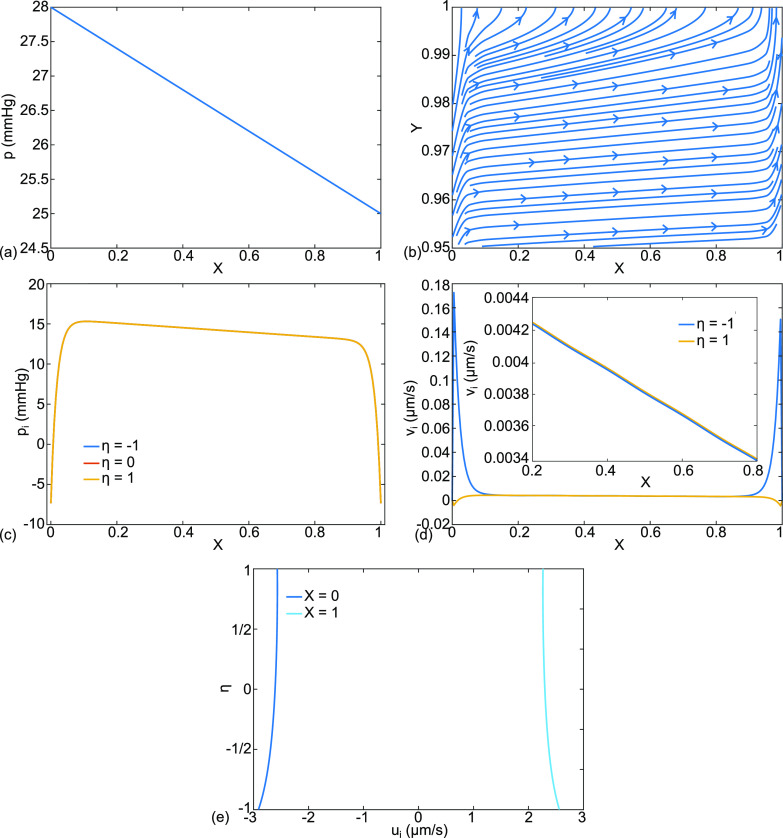
For cardiogenic edema, case (b), the base state parameter values have been modified for $p_{a}=28,\;\;p_{v}=25\;\mathrm{mmHg}$. The results are (a) dimensional capillary pressure p(X) in mm Hg, (b) dimensionless capillary velocity vector field (U,V) and streamlines for 0.95≤Y≤1.0, (c) interstitial fluid pressure $p_{i\;}(X,\eta =-1,0,1)$ in mm Hg, (d) alveolar membrane crossflow velocity, $v_{i\;}\left(\eta =1\right)$, and capillary membrane crossflow velocity, $v_{i\;}\left(\eta =-1\right)$, in *μ*m/s, and (e) X-velocity $u_{i\;}\left(X,\eta \right)$ for X=0,1, in *μ*m/s.

**FIG. 8.**
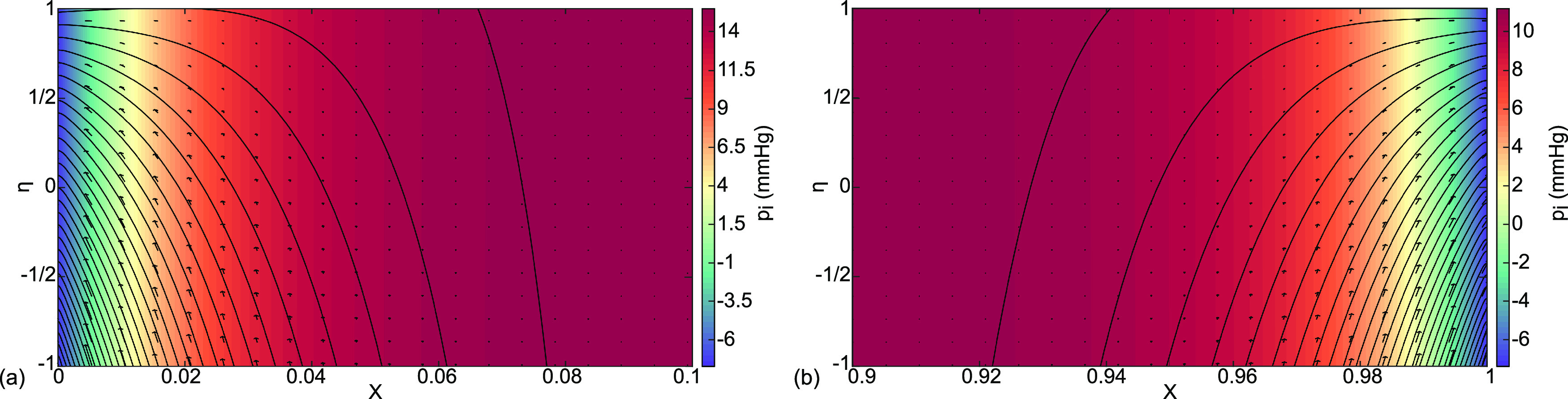
Case (b) interstitial velocity vector field $\left(U_{i\;},V_{i\;}\right)$, streamlines, and pressure distribution $p_{i}$ color coded in mm Hg for (a) the boundary layer at 0≤X≤0.1 and (b) the boundary layer at 0.9≤X≤1.

Case (b) in Figs. 7 and 8 is cardiogenic pulmonary edema, where capillary pressures p_a_ and p_v_ are elevated, while the other parameter values are held at base levels. A sketch of cardiogenic pulmonary edema is in Fig. 3(b), which shows flow driven into the alveolus by increased hydrostatic pressures. The capillary diameter is larger due to the pressure increase but not included for Fig. 7. Typically, this scenario is part of congestive heart failure.

**FIG. 9.**
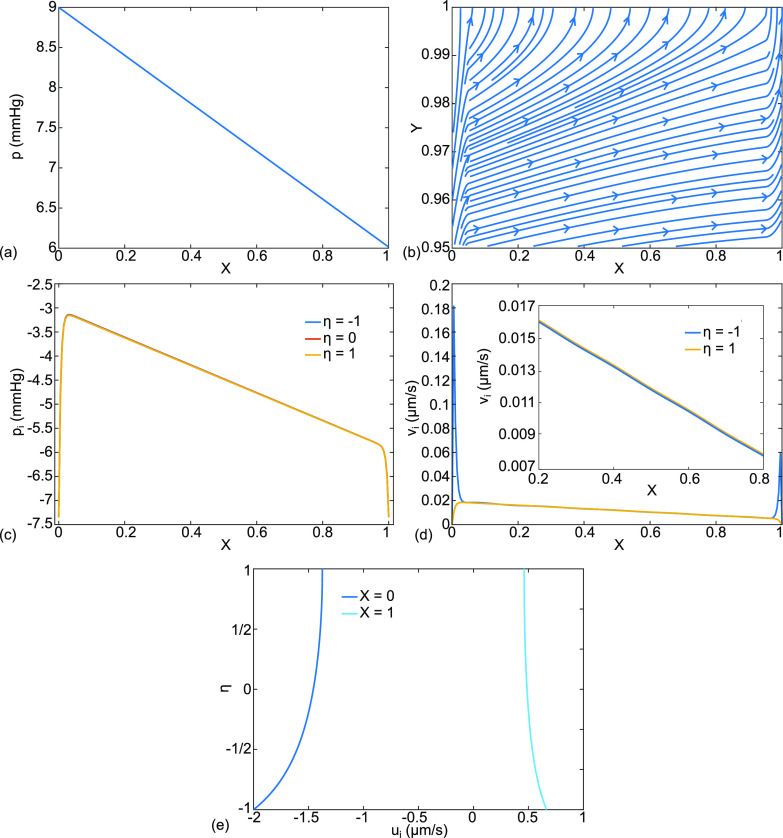
For ARDS, case (c), the base state parameter values have been modified for $p_{AL}=-7\;\;\mathrm{mmHg}$, $\pi_{AL}=10\;\mathrm{mmHg}$, $k_{c}=1\times 10^{-5}$, and $\;\;k_{A}=5\times 10^{-7}cm\cdot s^{-1}\cdot \mathrm{mmH}g^{-1}$ The results are (a) dimensional capillary pressure p(X) in mm Hg, (b) dimensionless capillary velocity vector field (U,V) and streamlines, (c) interstitial fluid pressure $p_{i\;}(X,\eta =-1,0,1)$ in mm Hg, (d) alveolar membrane crossflow velocity, $v_{i\;}\left(\eta =1\right)$, and capillary membrane crossflow velocity, $v_{i\;}\left(\eta =-1\right)$, in *μ*m/s, and (e) X-velocity $u_{i\;}\left(X,\eta \right)$ for X=0,1 in *μ*m/s.

**FIG. 10.**
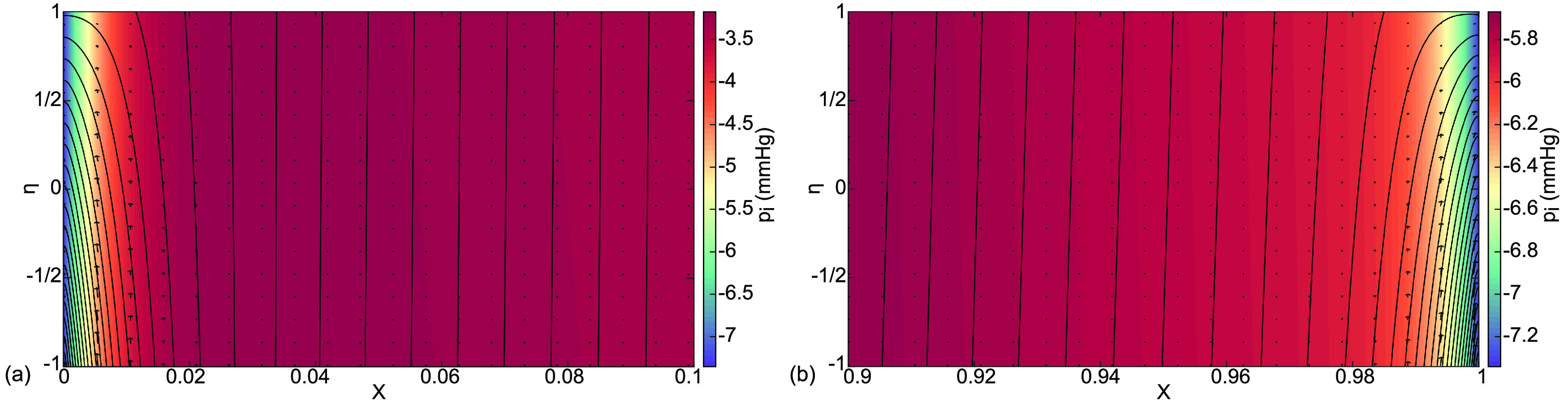
Case (c) interstitial velocity vector field $\left(U_{i\;},V_{i\;}\right)$, streamlines, and pressure distribution $p_{i}$ color coded in mm Hg for (a) the boundary layer at 0≤X≤0.1 and (b) the boundary layer at 0.9≤X≤1.

Noncardiogenic pulmonary edema of ARDS is studied in case (c), Figs. 9 and 10. A sketch of ARDS is in Fig. 3(c), which shows elements of inflammation involving macrophages and neutrophils. For COVID-19, lymphocytes are also prevalent.^94^ There are increased leaks (hydraulic conductivities) in both the capillary endothelium, k_c_, and alveolar membrane, k_A_. The alveolar edema fluid is rich with proteins, which cross through the reduced barrier and remain from inflammatory cellular breakdown. All three cases in Figs. 3(a)–3(c) show a region where there is flow across the interstitial layer and then a region where it transitions to flow out the ends of the interstitial layer to the lymphatics. That feature is captured in Figs. 6, 8, 10, and 12.

**FIG. 11.**
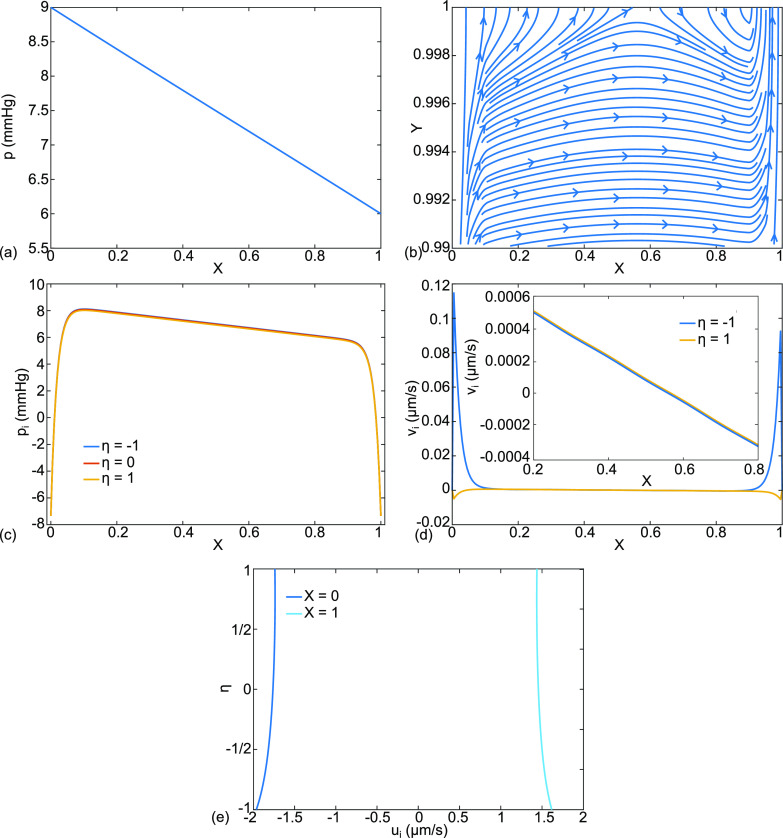
For case (d), hypoalbuminemia, the base state parameter values have been modified for low blood osmotic pressure, π=11  mmHg. The results are (a) dimensional capillary pressure p(X) in mm Hg, (b) dimensionless capillary velocity vector field (U,V) and streamlines, (c) interstitial fluid pressure $p_{i\;}(X,\eta =-1,0,1)$ in mm Hg, (d) alveolar membrane crossflow velocity, $v_{i\;}\left(\eta =1\right)$, and capillary membrane crossflow velocity, $v_{i\;}\left(\eta =-1\right)$ in *μ*m/s with inset, and (e) X-velocity $u_{i\;}\left(X,\eta \right)$ for X=0,1 in *μ*m/s.

**FIG. 12.**
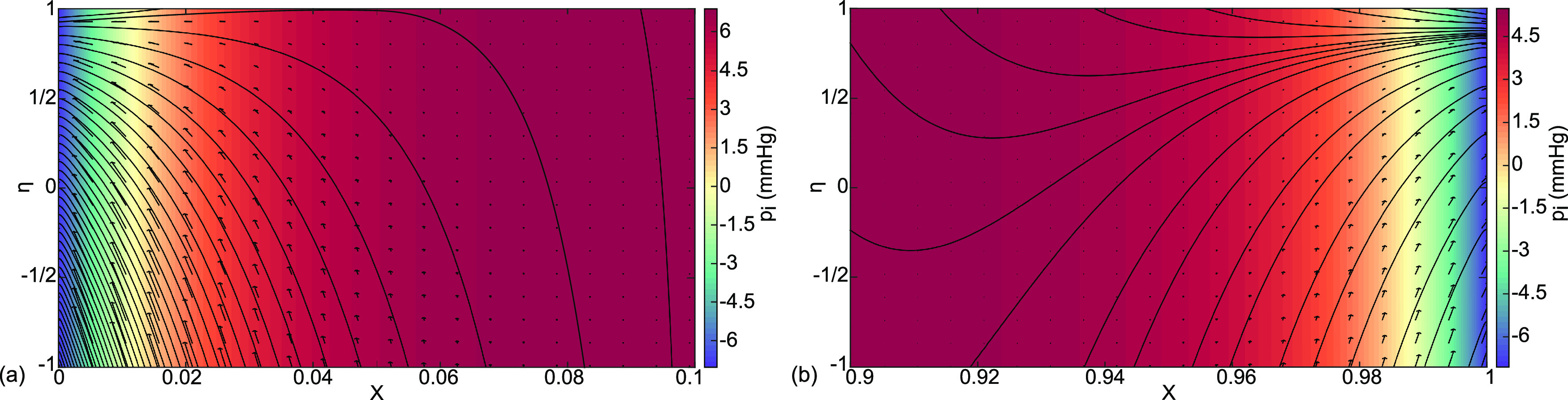
Case (d) hypoalbuminemia, interstitial velocity vector field $\left(U_{i\;},V_{i\;}\right)$, streamlines, and pressure distribution $p_{i}$ color coded in mm Hg for (a) the boundary layer at 0≤X≤0.1 and (b) the boundary layer at 0.9≤X≤1.

**FIG. 13.**
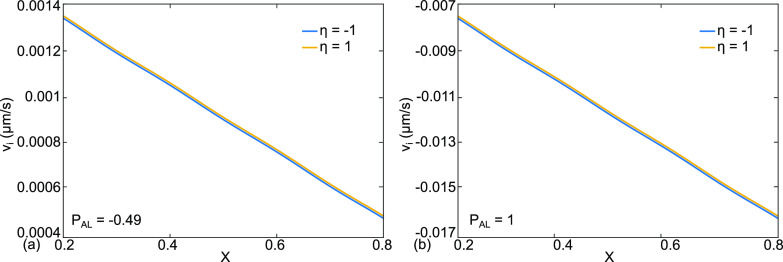
Case (e) is PEEP therapy for cardiogenic edema. The base state parameter values have been modified for p_AL_. The results for alveolar membrane crossflow velocity, $v_{i\;}\left(\eta =1\right)$, and capillary membrane crossflow velocity, $v_{i\;}\left(\eta =-1\right)$ in *μ*m/s for the base value (a) $p_{AL}=-1.47\;\;\mathrm{mmHg}$ and the PEEP value (b) $p_{AL}=3\;\;\mathrm{mmHg}$.

An essential contribution to the Starling equation is the capillary osmotic pressure, π, which is largely attributable to plasma proteins like albumin. Low levels, hypoalbuminemia, occur from reduced production by the liver, increased loss through the gastrointestinal tract or kidneys, and malnutrition. Case (d), Figs. 11 and 12, reduces π from the base value, leading to edema. An immediate therapeutic modality for pulmonary edema is to increase the alveolar gas pressure, p_AG_, using positive end expiratory pressure (PEEP), for example. This increases the alveolar liquid pressure, p_AL_, and can reverse the situation from edema to clearance as shown for case (e), Fig. 13, for cardiogenic edema.

Figure 4(a) shows normal cardiopulmonary physiology, case (a), using our base state parameter values from Nomenclature. The capillary dimensional pressure p(X) in Fig. 4(a) starts with the arterial value $p_{a}=9\;\mathrm{mmHg}$ at X = 0 and drops to the venous value $p_{v}=6\;\mathrm{mmHg}$ at X = 1. From the equation where $V(1)=(1/3)d^{2}P/dX^{2}$, the sign of V(Y=1) matches the sign of the curvature of P(X). In this case, both are slightly negative and the curvature is imperceptible in Fig. 4(a), where p(X) appears linear in X. Figure 4(b) shows the dimensionless capillary velocity field, (U,V), and streamlines in the magnified region 0.95≤Y≤1, indicating that fluid enters the capillary from the interstitium through the capillary membrane at Y=1. This is clearance to the capillaries. The crossflow displaces the streamlines away from the capillary boundary.

Figure 4(c) consists of the interstitial dimensionless velocity vector field, $\left(U_{i\;},V_{i\;}\right)$, streamlines, and dimensional pressure distribution $p_{i}$ with color coding in mm Hg. The horizontal variable has been transformed as ξ=λX, so that 0≤X≤1 covers the full length for both the capillary and the interstitium. Fluid crosses from the alveolar liquid, through the alveolar membrane at η=1 and into the interstitium. From there, nearly all of the fluid crosses the interstitium in the η-direction into the capillary. We call this the septal crossflow. The rest exits in the X-direction through the ends at X=0,1 as we will see below. Figure 4(d) shows more details for the interstitial pressure distribution at three values of η, $p_{i\;}(0\leq X\leq 1,\eta =-1,0,1)$, which for this case are overlapping. There is a central region, approximately 0.1≤X≤0.9 for this case, where p_I_ drops linearly in X down the length of the septal tract, from approximately −2.7 mm Hg to −5.0 mm Hg. However, in the end regions, approximately 0≤X≤0.1 and 0.9≤X≤1, there are steep decreases to match $p_{iB}$ at X = 0,1 respectively. We call these the boundary layers. The steep gradients are reflected in Fig. 4(c) where the color changes are compressed into the same boundary layers.

Figure 5 shows additional results for case (a) including Fig. 5(a), which plots the interstitial crossflow velocities at the alveolar membrane, $v_{i\;}\left(\eta =1\right)$, and the capillary membrane, $v_{i\;}\left(\eta =-1\right)$. There is an insert over the interval 0.2≤X≤0.8 to reveal the values and signs more clearly in the central region where the velocities are negative, showing flow from the alveolar liquid through the interstitium to the capillary. Both are essentially the same in the velocity range −0.005 to−0.006  μm/s, so whatever crosses one of the membranes crosses the other. In addition, they have negative slopes with increasing X, reflecting the capillary pressure drop. However, in the boundary layers, the velocities are much larger with opposite signs: positive across the capillary membrane and negative across the alveolar membrane. We see in Fig. 5(b) that these two streams crossing in opposite directions toward one another implies they turn the corner and exit together out the septal ends, see Fig. 6. As shown, outflow at the left end, X = 0, has the velocity range $-0.55<u_{i}(X=0)<-0.6\;\;\mu m/s$, and at the right end, X = 1, $0.24<u_{i}(X=1)<0.26\;\mu m/s$. We call these the septal tract end-flow velocities, also seen as the end vectors at X = 0,1 in Fig. 4(c). As indicated in Fig. 2, these flows are directed to the lymphatics.

The central region u_i_ velocities plotted in Fig. 5(c) for X=0.19,0.2, 0.4, 0.6, 0.8, and 0.81 are in the range 0.0058  μm/s with less than 1% changeover the dimensional distance 0.8 l. The overall x-gradient of u_i_ is approximated by $\Delta u_{i}/\Delta x=∼\left(5.8\times 10^{-5}\;\mu m/s\right)/\left(0.8\times 500\;\mu m\right)=1.45\times 10^{-7}\;s^{-1},$ which is essentially zero. From conservation of mass $\partial u_{i}/\partial x+\partial v_{i}/\partial y=0$, the implication is that this region has $\partial v_{i}/\partial y∼0$, implying that v_i_ is constant through the interstitial thickness away from the boundary layers. That observation is consistent with Fig. 5(a). Since the capillary pressure drop over L is $p_{a}-p_{v}=3\;\;\mathrm{mmHg}$, the interstitial pressure drop, for the interior region 0.1<X<0.9 is similar, $\Delta p_{i}∼3\;\mathrm{mmHg}$. An average X-velocity in this interior, using Darcy Law, is ${\bar{u}}_{i}=K\left(1333\right)\Delta p_{i}/\mu_{i}L=6.15\times 10^{-3}\mu m/s$ in the positive X-direction, which falls in the range of Fig. 5(c). All of the cases (a)–(e) presented have the same capillary pressure drop and the same values of $K,\;\mu_{i}\;,\;\;L$, and consequently similar interior ${\bar{u}}_{i}$.

Figures 5(d) and 5(e) show the velocities and pressures in the boundary layers. There are rapid changes of both $p_{i\;}(\eta =0)$, as we saw in Fig. 4(d), and $u_{i\;}(X,\eta =0)$ in the regions 0≤X≤0.1 and 0.9≤X≤1 to match the pressure boundary condition $p_{i\;}=p_{iB}=-7.35\;\mathrm{mmHg}$ at the ends, X = 0,1, where it is imposed. In the central region, $p_{i}$ is significantly more positive than p_iB_, contrary to traditional physiological assumptions that $p_{i\;}=p_{iB}$ everywhere in the interstitium.

Expanded views of the interstitial boundary layers in Fig. 4(c) are shown in Fig. 6. In Fig. 6(a), there are streamlines closer to X = 0 that curve to exit to the lymphatics. They come from both capillary and alveolar membranes, η=−1, 1 respectively, which gives $v_{i}(\eta =-1)>0$ and $v_{i}(\eta =1)<0$. In this region, the lymphatics would be clearing fluid from the alveolus while also diverting fluid from the capillary away from the alveolus. Farther from X = 0, the streamlines are straight with flow from η=1 to η=−1, clearance from the alveolus to the capillary. These patterns are consistent with the velocities of Fig. 5(a) and the general flow directions of Fig. 3. There, however, the process is sketched over a single alveolus, as opposed to the present model that covers the alveolar capillary coursing past several alveoli. At the other end, Fig. 6(b) also shows streamlines from both the capillary and the alveolar membranes exiting to the lymphatics together. However, because the capillary pressure is lower at this downstream location, the capillary contribution is less. So, the protective role of these end flows is both to remove fluid from the lung while also diverting capillary fluid toward the lymphatics.

An example of cardiogenic edema is shown in Fig. 7, case (b), by using our base parameter set, but with elevated capillary pressures $p_{a}=28\;,\;\;p_{v}=25\;\;mm\;Hg$. These values fall in the range used in isolated, perfused dog lungs to study pulmonary hypertension and edema formation.^95^ Figure 7(a) shows the capillary pressure, p, while Fig. 7(b) plots streamlines and velocity vectors for fluid that exits the capillary and enters the interstitium, opposite to case (a) in Fig. 4(b). This is pulmonary edema. The interstitial fluid pressure, Fig. 7(c), again shows boundary layers where there are rapid decreases in p_i_ to match p_iB_ at the ends. For the central region, p_i_ drops linearly with X in the range $13\leq p_{i}\leq 16\;\;\mathrm{mmHg}$, approximately. The crossflows at the capillary and alveolar membranes in Fig. 7(d) are essentially equal outside of the boundary layers, as in case (a). Both are positive in the range 0.0034−0.0042  μm/s, so in this central region, fluid leaves the capillary, crosses the interstitium, and enters the alveolar compartment. The septal end velocities are $-2.5<u_{i}(X=0)<-3$ and $2.2<u_{i}(X=1)<2.6\;\;\mu m/s$, see Fig. 7(e). These are much larger values compared to case (A), reflecting the much larger pressure jump at the ends.

A closer look at the boundary layer regions, the velocities, and streamlines in Fig. 8 indicate that the overwhelming contribution to the end flows comes from the capillaries with almost none from the alveoli. So, the lymphatics receive fluid diverted from the blood flow as a protective mechanism for the alveoli.

Case (c) is representative of edema from ARDS, see Fig. 9. Our base parameter set is modified to increase both $k_{c},\;k_{A}$ by a factor of 10 for greater permeability of the capillary and alveolar membranes; decrease $p_{AL}$ to be more negative, reflecting inactivation of surfactant with higher surface tension; and, increase $\pi_{AL}$ for increased proteinaceous material in the alveolar liquid. Accordingly, the new parameter values are $k_{c}=1\times 10^{-5}$, $k_{A}=5\times 10^{-7}cm\cdot s^{-1}\cdot \mathrm{mmH}g^{-1}$, $p_{AL}=-7\;\mathrm{mmHg}$, and $\pi_{AL}=10\;\;\mathrm{mmHg}$. Like case (b), fluid crosses from the capillaries through the interstitium and into the alveolar liquid, see Figs. 9(b) and 9(d), i.e., pulmonary edema. The boundary layers are thinner than cases (a) and (b), and septal crossflow velocity ranges from $0.008\leq v_{i}\leq 0.016\;\;\mu m/s$ in the central region. The interstitial pressure, $p_{i}$, decreases linearly in the central region over a range $-3.2\leq p_{i}\leq -5.8\;\;\mathrm{mmHg}$ in Fig. 9(c), again reflective of the capillary pressure drop. It is noteworthy that these values of p_i_ are very similar to the normal in case (a). We show later that p_i_ depends on the ratio $\left(k_{A}/k_{c}\right)$, which is the same for case (c) and case (a), since both k_A_ and k_c_ are increased by a factor of 10 in case (c). The septal end-flow velocities of Fig. 9(e) are $-1.4<u_{i}(X=0)<-2.0$ and $0.4<u_{i}(X=1)<0.7\;\mu m/s$.

The thinner boundary layer flow details are shown in Fig. 10. As with cardiogenic pulmonary edema, case (b), the flow is from the capillary to the alveolus, except close enough to the end boundary where the streamlines curve out through the exit to the lymphatics. Again, the protective function is to divert flow from the capillaries away from the alveolar compartment.

Case (d) consists of low blood osmotic pressure shown in Fig. 11. This situation can occur clinically with hypoalbuminemia,^95^ which has been investigated by others.^96^ Different from the previous cases, the value of π=11 mmHg. The results for the capillary velocities and streamlines, Fig. 11(b), show flow from the capillary into the alveolus upstream, but then flow from the alveolus to the capillary downstream. So only a part of the septal tract experiences pulmonary edema, while the rest is being cleared. This value of π was specifically chosen to give this mixed result. A similar reversal could be achieved, for example, with normal π but with $p_{a}=20$ and $p_{v}=17\;\;\mathrm{mmHg}$. The interstitial fluid pressure, $p_{i}$, drops linearly with X, from 8 to 6 mm Hg, in the central region, Fig. 11(c), even though the capillary pressures are normal, Fig. 11(a). The boundary layers are steeper due to the higher positive values of p_i_. Figure 11(d) shows the details of v_i_ with the crossover point, X ∼ 0.55, where v_i_ switches from positive to negative. The septal end-velocities of Fig. 11(e) vary over the range $-1.75\leq u_{i}(X=0,\eta )\leq -2$ and $-1.75\leq u_{i}(X=0,\eta )\leq -2\;\mu m/s$.

The boundary layer flow details in Fig. 12 also reflect the switch from upstream edema to downstream clearance. The streamlines in Fig. 12(a) originate from the capillary and exit at X = 0. However, at the other end, X = 1, the boundary layer in Fig. 12(b) shows a mix of streamlines from both the alveolus and the capillary, where the blood pressure is lower than that at the X = 0 end. The combined flows protect the alveolus with both clearance and diverting of capillary flow.

The model can also be used to explore and understand therapy. Figure 13, case (e), is another example of cardiogenic edema, less severe than case (b). The vascular pressures are $p_{a}=22$ and $p_{v}=19\;\;\mathrm{mmHg}$. Figure 13(a) shows the alveolar membrane crossflow velocity, $v_{i\;}\left(\eta =1\right)$, and capillary membrane crossflow velocity, $v_{i\;}\left(\eta =-1\right)$ in μm/s for the base alveolar liquid pressure $p_{AL}=-1.47\;\mathrm{mmHg}$. The result is pulmonary edema. However, in Fig. 13(b), the alveolar gas pressure is increased so that $p_{AL}=3\;\;\mathrm{mmHg}$. This may be done using PEEP with a ventilator, BiPAP (Bilevel positive airway pressure), or CPAP (continuous positive airway pressure), and now the crossflow is reversed, stopping the edema and promoting clearance. Raising p_AL_ through p_AG_ is an example of potentially patient-specific therapies, since the effect of PEEP will also depend on the other parameters in the model, all of which can vary from patient to patient. The model provides a framework to sort out which therapies are available and adjustable by exploring which parameters are the most influential and can also be measured.

## DISCUSSION

Our microvascular model of lung interstitial fluid transport solves the pressure and velocity fields of the coupled system for capillary (lubrication theory), interstitial (Darcy flow), and alveolar (passive) compartments. In addition to mass and momentum conservation within each compartment, boundary conditions of fixed pressures at the ends (X = 0,1) and the Starling equation at both the capillary-interstitium boundary and alveolar-interstitium boundary are imposed. The system is solved using Fourier series, so is available for potential users. The calculations are only for the initial fluid mechanical response. They do not include tissue compliance, active fluid and solute transport,^57,58^ lymphatics,^97^ constraints on alveolar liquid supply, dependence of parameters on one another (e.g., pressure dependent K, d, and b), adaptations over time, three dimensional effects, or respiratory motions.^98^

Case (a) shows fluid exiting the alveolus and entering the capillary over the interior of the septal tract, 0.01≤X≤0.99, while flow is in the opposite direction for pulmonary edema in cases (b) and (c) and bidirectional for case (d). The use of PEEP to reverse edema is shown in case (e). For all of the figures, the decreasing capillary pressure, p, downstream causes a downstream drop in the interstitial pressure, $p_{i}$. So bidirectional flow could occur in any of the edema cases, not just case (d), with convection into the alveolus upstream and out of the alveolus downstream. Such situations may be a source of different levels of clinical and radiographic findings. For example, inspiratory crackles are lung sounds used to monitor pulmonary edema and have been implicated in causing injury to small airways.^99–104^ The crossflow velocities at the alveolar and capillary membranes are essentially equal in the interior.

As part of the analysis, the interstitial pressure, $p_{i}(x,y\prime )$, is calculated and found to be very different from traditional values used in lung physiology. To our knowledge, this is the first calculation of $p_{i}$ from a detailed fluid mechanics model. Because of the absence of direct measurements, the model's computations can help to establish a foundation for understanding and interpreting basic lung physiology and pathophysiology. Micropipette pressure measurements in nearby interstitial tissue^66,67^ were imposed as the end pressures $p_{iB}=-7.35\;\;\mathrm{mmHg}$. This value is similar to indirect estimates of a constant $p_{i}$,^105^ −9, and −8 to−7 mmHg.^91^ Others have proposed more positive values of −5–0 cmH_2_O.^97^ However, the only way for $p_{i}=p_{iB}$ everywhere in the model is to make both the alveolar and capillary membranes impermeable by setting $k_{c}=k_{A}=0$. These values force $a_{n}=b_{n}=f_{n}=0$, so only the constant, $P_{iB}$, term survives in Eq. (9). Consequently, because of the Darcy flow model, there is no interstitial fluid velocity $\left(u_{i},v_{i}\right)=-\left(K/\mu \right)\left(\partial p_{i}/\partial x,\partial p_{i}/\partial y^{\prime }\right)=\left(0,0\right)$ under these conditions.

What we see, instead, is that $p_{i}$ is much more positive than $p_{iB}$, which leads to septal tract end-flows in the boundary layer regions. This is a new physiological flow phenomenon. Under traditional views that $p_{i}=p_{iB}$ everywhere, such a flow would not be possible. The septal end-flow velocities can be relatively large and may include contributions from both the capillary and alveolar liquid.

In the discussion of Fig. 5(c), the central region can be considered to be locally one-dimensional flow where v_i_ is a constant through the interstitial layer as is the vertical pressure gradient. Let $v_{i}=k_{i}\left(p_{ic}-p_{iA}\right)$, where pressures are expressed in mm Hg. Then, $k_{i}=1333\;\;K/2d\mu_{i}$, $p_{ic}=p_{i}(y^{\prime }=-d)$, and $p_{iA}=p_{i}(y\prime =d)$. This system can be viewed as three flow resistors in series. The Darcy resistor is sandwiched between the alveolar and capillary membrane resistors. Now equate the Darcy v_i_ to both expressions of v_i_ for the alveolar and capillary membrane Starling equations,

$$\begin{array}{c} k_{i}\left(p_{ic}-p_{iA}\right)=k_{A}\left(p_{iA}-P_{A}\right)\\ k_{i}\left(p_{ic}-p_{iA}\right)=k_{c}\left(-p_{ic}-P_{c}\right) \end{array},$$ (1)

where $P_{A}=p_{AL}+\sigma_{A}\left(\pi_{i}-\pi_{AL}\right)$ and $P_{c}=-p+\sigma_{c}(\pi_{c}-\pi_{i})$. The solutions to Eq. (1) for p_ic_ and p_iA_ are

$$\begin{array}{c} p_{ic}=\frac{-k_{c}P_{c}\left(k_{A}+k_{i}\right)+k_{i}P_{A}k_{A}}{\left(k_{c}+k_{i}\right)k_{A}+k_{i}\;k_{c}},\\ p_{iA}=\frac{k_{A}P_{A}\left(k_{c}+k_{i}\right)-k_{i}\;P_{c}\;k_{c}}{\left(k_{c}+k_{i}\right)k_{A}+k_{i}\;k_{c}}. \end{array}$$ (2)

For our parameter choices, $k_{i}=1.28\times 10^{-4}\;cm\cdot \mathrm{mmH}g^{-1}\cdot s^{-1}$, which is 2–3 orders of magnitude larger than k_c_ and k_A_ in Nomenclature. Taking the limit of Eq. (2) for $k_{i}\gg k_{c},\;k_{A}$ yields $p_{ic}∼p_{iA}=p_{i}$,

$$p_{i}=\frac{\left(p-\sigma_{c}(\pi_{c}-\pi_{i})\right)+\left(k_{A}/k_{c}\right)\left(p_{AL}+\sigma_{A}\left(\pi_{i}-\pi_{AL}\right)\right)}{1+\left(k_{A}/k_{c}\right)},$$ (3)

which is independent of k_i_, while k_c_ and k_A_ only appear as the ratio $\left(k_{A}/k_{c}\right)$. This explains why p_i_ for case (a) and case (c) are nearly the same, since the two values are increased by a factor of 10 in case (c). In Eq. (4), the interstitial osmotic pressure, $\pi_{i}$, drops out when $\sigma_{A}=\sigma_{c}$, as assumed for our analysis.

Substituting Eq. (4) into the capillary Starling equation gives the velocity, v_i_,

$$v_{i}=k_{A}k_{c}\left(\frac{p-p_{AL}+\sigma_{A}\left(\pi_{AL}-\pi_{i}\right)+\sigma_{c}(\pi_{i}-\pi_{c})}{\left(k_{A}+k_{c}\right)}\right).$$ (4)

**FIG. 14.**
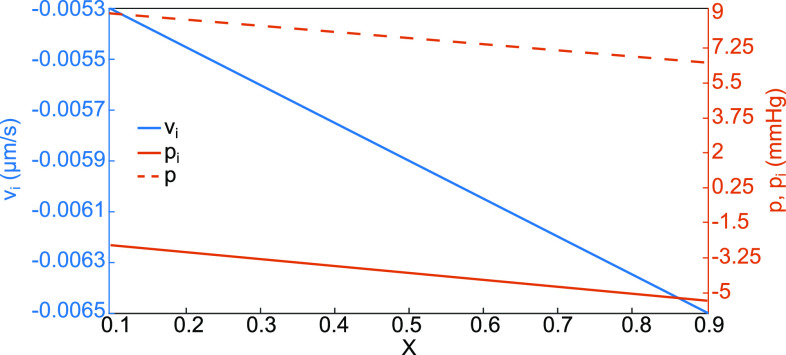
p, p_i_, and v_i_ in the central region 0.1≤X≤0.9 using the locally one-dimensional analysis.

The locally one-dimensional solutions for the interstitial pressure, p_i_, and velocity, v_i_, can be plotted vs X assuming p(X) decreases linearly, see Fig. 14. We recognize these approximate solutions represent well the central regions of Fig. 4(d) for pressure and Fig. 5(a) for velocity.

When $v_{i}>0$, the flow promotes edema, but when $v_{i}<0$, the flow promotes clearance. The critical value of the capillary pressure, $p_{\text{crit}}$, is when $v_{i}=0$, yielding

$$p_{\text{crit}}=p_{AL}+\sigma_{A}\left(\pi_{i}-\pi_{AL}\right)+\sigma_{c}(\pi_{c}-\pi_{i}),$$ (5)

which is independent of k_A_ and k_c_. Then, $p>p_{\text{crit}}$ causes $v_{i}>0$, and $p<p_{\text{crit}}$ causes $v_{i}<0$. For our base state parameter set $p_{\text{crit}}=19.85\;\mathrm{mmHg}$, which is consistent with clinical settings as demonstrated in cases (b) and (e). In Eq. (5), we can replace p_AL_ with the law of Laplace, $p_{AL}=\left(p_{AG}-2\sigma /R\right)$, to obtain

$$p_{\text{crit}}=\left(p_{AG}-2\sigma /R\right)+\sigma_{A}\left(\pi_{i}-\pi_{AL}\right)+\sigma_{c}(\pi_{c}-\pi_{i}).$$ (6)

It is protective to increase p_crit_ by increasing the alveolar gas pressure, p_AG_, as seen in case (e) involving PEEP or decreasing surface tension, σ, say with surfactant therapy.^33–35^ For ARDS, we lowered p_AL_ due to increased σ, which contributed to edema. Using the Laplace's law in Eq. (3) permits a calculation of interstitial pressure,

$$p_{i}=\frac{\left(p-\sigma_{c}(\pi_{c}-\pi_{i})\right)+\left(k_{A}/k_{c}\right)\left(\left(p_{AG}-2\sigma /R\right)+\sigma_{A}\left(\pi_{i}-\pi_{AL}\right)\right)}{1+\left(k_{A}/k_{c}\right)}.$$ (7)

Since alveolar capillary pressure, p, is estimated clinically using pulmonary catheter wedge pressures and p_AG_ is a ventilator setting, Eq. (7) can be used to estimate the interstitial pressure. Traditionally, p_i_ is considered as an input, since only the capillary membrane Starling equation is used. Here, we see it is not only an output but can be calculated from available measurements.

The puzzle of how the lymphatics can function so far away from the alveoli has captivated lung physiologists and anatomists dating back to 1896.^12,97,106,107^ The early quest was to find lymphatics much closer, next to the alveolar walls, but they were not there. As pointed out by Staub,^97^ Tobin's^107^ comment captures the frustration: “this concept (no alveolar wall lymphatics) has made it difficult to understand by what means foreign material or fluid in the alveoli may be transported to the nearest lymphatics.” As Staub says, “the problem posed by Tobin remains and demands a rational answer.” One possible explanation is the septal tract end-flow in our model, i.e., interstitial pressure created by the coupled compartments drives its own clearance. This is a new physiological flow.

## METHODS

Figure 2 (left) is a two-dimensional model of a septal tract with three compartments: capillary, interstitium, and alveolus. The assumption of two dimensions is well justified since the alveolar capillary system geometry is often considered as flow between parallel alveolar sheets.^50,51^ There is normally a thin liquid layer coating the inside surface of an alveolus. The surface tension, σ, between it and the alveolar gas is reduced by surfactants produced by alveolar epithelial type II cells.

Four alveoli are represented with alveolar gas pressure p_AG_, liquid pressure p_AL_, and surface tension, σ, at the gas–liquid interface, and osmotic pressure, π_AL,_ Fig. 2 (right) shows the radius, R, of the alveolus. The upper half of the capillary is bounded by 0≤x≤L ,0≤y≤b, while the top interstitium strip occupies $0\leq x\leq L\;,-d\leq y^{\prime }\leq d$. Note that the capillary-interstitial boundary occurs at $y^{\prime }=-d$, which is the same as y=b. The lower half of the capillary and the lower interstitium strip, not shown, will be the mirror image of the upper halves, so there is symmetry of the entire system with respect to the capillary centerline, y = 0.

Capillary flow is driven by pressure differences between the upstream arterial, p_a_, and downstream venous, p_v_, ends. That motion is governed by the Navier–Stokes equations, which we simplify for thin layers using lubrication theory. Flow in the interstitium obeys Darcy's equations, appropriate for a porous media, and the alveolar liquid is passive.

The capillary membrane at $y=b\;\text{and}\;y^{\prime }=-d$ is semipermeable, allowing fluid crossflows, in either direction, between the capillary blood and the interstitial fluid. Likewise, the semipermeable alveolar membrane at $y^{\prime }=d$ allows crossflows between the interstitium and the alveolar liquid.

Pulmonary edema occurs when fluid crosses into the alveolus at a rate faster than it can be cleared. Flows to the lymphatics leave through the left hand and right hand ends of the interstitial tract, as indicated by blue arrows. The mathematical analysis involves solving for the conservation of mass and momentum for the capillary blood flow, where the pressure is p, the X-velocity is u, and the Y-velocity is v. In addition, mass and momentum are conserved for the interstitium, where the pressure is p_i_, the X-velocity is u_i_, and the Y′-velocity is v_i_. The Starling equation is applied at both the capillary membrane and the alveolar membrane, and the tract ends have an applied pressure, p_iB_.

### Capillary blood flow

Using the parameter and variable definitions in Nomenclature, the dimensionless X-velocity, U, and pressure, P, satisfy the dimensionless Navier–Stokes equation simplified for lubrication theory,

$$-\frac{\partial P}{\partial X}+\frac{\partial^{2}U}{\partial Y^{2}}=0,\;\;\;\;\frac{\partial P}{\partial Y}=0.$$ (8)

The coordinate value ranges are 0≤X≤1 ,−1≤Y≤1. Eq. (8) assumes ε=b/L≪1 and εRe≪1, where Re is the capillary Reynolds number which, itself, satisfies Re≪1. From the Y-component of Eq. (8), we learn that P is independent of Y, so P=P(X). Integrating the X-component of Eq. (8) leads to the solution form of U,

$$U=-\frac{1}{2}\frac{dP}{dX}\left(1-Y^{2}\right),$$ (9)

where U(Y=±1) satisfies no-slip at the capillary membranes, and we see that U is locally parabolic.

Conservation of mass is given by ∂U/∂X+∂V/∂Y=0, which allows us to solve for the Y-velocity, V. Enforcing no crossflow at the centerline, V(Y=0)=0, due to symmetry V is

$$\;V=-\int_{0}^{Y}\frac{\partial U}{\partial X}\;dY=\frac{1}{2}\frac{d^{2}P}{dX^{2}}\left(Y-\frac{Y^{3}}{3}\right).$$ (10)

### Interstitium

Modeled as a porous media, the interstitium has permeability, K, also known as the specific hydraulic conductivity,^52^ and fluid viscosity, $\mu_{i}$. The ratio $K\;/\mu_{i}$ is termed the hydraulic conductivity, and its inverse is the hydraulic resistance. Conservation of momentum is given by the dimensionless form of the Darcy equations,

$$U_{i}=-\frac{\partial P_{i}}{\partial \xi }\;,\;V_{i}=-\frac{\partial P_{i}}{\partial \eta },$$ (11)

where $U_{i}\;,\;V_{i}$ are the dimensionless velocities in the ξ , η directions, respectively, and $P_{i}$ is the dimensionless pressure. The coordinate value ranges are 0≤ξ≤λ,−1≤η≤1. The conservation of mass equation is $\partial U_{i}/\partial \xi +\partial V_{i}/\partial \eta =0$, which, after inserting Eq. (11), yields the Laplace's equation for $P_{i}$,

$$\frac{\partial^{2}P_{i}}{\partial \xi^{2}}+\frac{\partial^{2}P_{i}}{\partial \eta^{2}}=0.$$ (12)

The crossflow at the permeable capillary membrane is given by the Starling equation^53^ in dimensional form $v(y=b)=k_{c}\left(p-p_{i}(y^{\prime }=-d)-\sigma_{c}(\pi -\pi_{i})\right)$, where k_c_ is the hydraulic conductivity, often denoted by L_p_ in physiological literature. The osmotic pressures operate in the opposite direction as the hydraulic pressures. The larger osmotic pressure side draws fluid away from the lower osmotic pressure side, hence the negative sign in front of the reflection coefficient, $\sigma_{c}$. The value of $0\leq \sigma_{c}\leq 1$, gauges the ability of osmotically active molecules, like albumin or other plasma proteins, to cross the membrane. For $\sigma_{c}=1$, those molecules do not cross, while a value of $\sigma_{c}=0$ indicates a freely permeable boundary with no effective osmotic pressure difference. The Starling model has further revisions to account for specific issues,^27,54–56^ which we do not address. Using the dimensionless variables, substituting for $V(Y=1)=(1/3)\;\left(d^{2}P/dX^{2}\right)$ from Eq. (10), Starling's equation becomes a differential equation for P coupled to $P_{i}$ in the ξ variable,

$$\frac{d^{2}P}{d\xi^{2}}-\kappa_{c}^{2}P=\kappa_{c}^{2}\left(-P_{i\;}(\eta =-1)-S_{c}\right).$$ (13)

We also need to match the crossflow velocities at the endothelial boundary, $v_{i}(y^{\prime }=-d)=v(y=b)$. In dimensionless form, this condition is

$$V_{i\;}(\eta =-1)=\beta V(Y=1).$$ (14)

The permeable alveolar membrane boundary condition is also modeled using Starling's equation $v_{i}(y^{\prime }=d)=k_{A}\left(\left(p_{i}(y^{\prime }=d)-p_{AL}\right)-\sigma_{A}\left(\pi_{i}-\pi_{AL}\right)\right)$, where k_A_ is the hydraulic conductivity, while p_AL_ and $\pi_{AL}$ are the alveolar liquid and osmotic pressures, respectively. This membrane also has active transport processes for water and salt,^57,58^ which can help to resolve pulmonary edema over time. We will not model active transport features in the current model. In dimensionless form, the flux across the alveolar membrane is

$$V_{i\;}(\eta =1)=-{\frac{\partial P_{i\;}}{\partial \eta }|}_{\eta =1}=\kappa_{A}^{2}\left(\left(P_{i\;}(\eta =1))-P_{AL}\right)-S_{A}\right).$$ (15)

### Solution

We solve the above system of equations and boundary conditions using Fourier analysis. For interstitial end boundary pressures, $P_{i\;}=P_{iB}$ at ξ=0,λ, let the leading order pressure field be given by the Fourier sine series

$$P_{i}=P_{iB}+\sum_{n=1}^{N}f_{n}(\eta )\;\text{sin}\;\left(k_{n}\xi \right),$$ (16)

where $k_{n}=n\pi /\lambda$, where N = 2000 in our computations. Inserting Eq. (16) into Eq. (12) yields a differential equation for $f_{n}(\eta )$, $f_{n}^{\prime \prime }-k_{n}^{2}f_{n}=0$, whose general solution is $f_{n}=a_{n}e^{k_{n}\eta }+b_{n}e^{-k_{n}\eta }$. Using Eq. (13) and substituting for $P_{i\;}(\eta =-1)$ from Eq. (16) yields

$$\frac{d^{2}P}{d\xi^{2}}-\kappa_{c}^{2}\;P=-\kappa_{c}^{2}\left(P_{iB}+\sum_{n=1}^{\infty }\left(a_{n}e^{-k_{n}}+b_{n}e^{k_{n}}\right)\text{sin}\;\left(k_{n}\xi \right)\right)-\kappa_{c}^{2}S_{c},$$ (17)

switching to the ξ variable for both sides where ξ=λX. The solution to Eq. (18), now in terms of X, is given by

$$P=\left(c_{0}e^{\kappa_{c}\lambda X}+d_{0}e^{-\kappa_{c}\lambda X}\right)+S_{c}+P_{i\;B}+\sum_{n=1}^{\infty }c_{n}\;\text{sin}\;\left(n\pi X\right),$$ (18)

where the first bracketed term is the homogeneous solution, involving the coefficients $c_{0}\;,\;d_{0}$, and the rest is the particular solution. By inserting Eq. (18) into Eq. (17) and equating coefficients of sin (nπX), the values of c_n_ are found to be $c_{n}=\kappa_{c}^{2}\left(a_{n}e^{-k_{n}}+b_{n}e^{k_{n}}\right)/\left(k_{n}^{2}+\kappa_{c}^{2}\right)$. Imposing the upstream and downstream blood pressure conditions on Eq. (18), $P(X=0)=P_{a}\;,\;\;\;P(X=1)=P_{v}$, determine $c_{0}=\left(\left(P_{iB}+S_{c}-P_{a}\right)e^{-\kappa_{c}\lambda }+\left(-P_{iB}-S_{c}+P_{v}\right)\right)/\left(e^{\kappa_{c}\lambda }-e^{-\kappa_{c}\lambda }\right)$ and $d_{0}=-\left(\left(P_{iB}+S_{c}-P_{a}\right)e^{\kappa_{c}\lambda }+\left(-P_{iB}-S_{c}+P_{v}\right)\right)/\left(e^{\kappa_{c}\lambda }-e^{-\kappa_{c}\lambda }\right)$.

The solutions for U, V, come from substituting for P, from Eq. (18) into Eqs. (9) and (10),

$$\begin{array}{c} U=-\frac{1}{2}\left(1-Y^{2}\right)\left(\kappa_{c}\lambda c_{0}e^{\kappa_{c}\lambda X}-\kappa_{c}\lambda d_{0}e^{-\kappa_{c}\lambda X}+\sum_{n=1}^{\infty }n\pi c_{n}\;\text{cos}\;\left(n\pi X\right)\right),\\ V=\frac{1}{2}\left(Y-\frac{Y^{3}}{3}\right)\left({\left(\kappa_{c}\lambda \right)}^{2}c_{0}e^{\kappa_{c}\lambda X}+{\left(\kappa_{c}\lambda \right)}^{2}d_{0}e^{-\kappa_{c}\lambda X}-\sum_{n=1}^{\infty }n^{2}\pi^{2}c_{n}\;\text{sin}\;\left(n\pi X\right)\right). \end{array}$$ (19)

Substituting Eqs. (11) and (16) into the left hand side of Eqs. (14) and (19) into the right hand side of Eq. (14) yields an equation involving the coefficients a_n_ and b_n_. Further manipulation is needed to express the exponentials in their own sine series: $e^{\kappa_{c}\xi }=\sum_{n=1}^{\infty }g_{n}\;\text{sin}\;\left(k_{n}\xi \right)$, where $g_{n}=2n\pi \left(1-e^{\kappa_{c}\lambda }{\left(-1\right)}^{n}\right)/\left(\pi^{2}n^{2}+\kappa_{c}^{2}\lambda^{2}\right)$ and $e^{-\kappa_{c}\xi }=\sum_{n=1}^{\infty }h_{n}\;\text{sin}\;\left(k_{n}\xi \right)$, where $\;h_{n}=2n\pi \left(1-{\left(-1\right)}^{n}e^{-\kappa_{c}\lambda }\right)/\left(\pi^{2}n^{2}+\kappa_{c}^{2}\lambda^{2}\right)$. Collecting the coefficients of $\text{sin}\;\left(k_{n}\xi \right)$ yields

$$-\left(k_{n}a_{n}e^{-k_{n}}-k_{n}b_{n}e^{k_{n}}\right)=\frac{\beta }{3}\left({\left(\kappa_{c}\lambda \right)}^{2}c_{0}g_{n}+{\left(\kappa_{c}\lambda \right)}^{2}d_{0}h_{n}-n^{2}\pi^{2}c_{n}\right).$$ (20)

Substituting for P_i_ from Eq. (16) into Eq. (15) gives us a second equation for $a_{n}\;,\;b_{n}$ and, again, we need to express all terms in the form of sine series. Let $\kappa_{A}^{2}\left(P_{iB}-P_{AL}-S_{A}\right)=\sum_{n=1}^{\infty }d_{n}\;\text{sin}\;\left(k_{n}\xi \right)$, where $d_{n}=-2\left(\kappa_{A}^{2}\left(P_{iB}-P_{AL}-S_{A}\right)\left({\left(-1\right)}^{n}-1\right)\right)/\left(n\pi \right)$. Now all of the terms multiply $\text{sin}\;\left(k_{n}\xi \right)$, and we can equate the coefficients

$$-k_{n}\left(a_{n}e^{k_{n}}-b_{n}e^{-k_{n}}\right)-\kappa_{A}^{2}\left(a_{n}e^{k_{n}}+b_{n}e^{-k_{n}}\right)=d_{n}.$$ (21)

The two equations, Eqs. (20) and (21), are solved for the remaining two unknown coefficients, $a_{n}\;,\;b_{n}$.

### Parameter values

The Navier–Stokes equations are simplified by lubrication theory assuming the capillary channel height is much smaller than its length, 2b/L≪1. A typical capillary diameter, 2b, has a range of 6.3–8.3 *μ*m,^59,60^ while its length, L, continues past several alveoli, the path length through the alveolar capillary network ranges from 250 to 850 *μ*m in several mammalian species.^6–8^ For our base state, we choose L=500, b=3 μm, so that ε=0.006. The total interstitial thickness, 4d, across the alveolar septum has been measured over a range, 1.63±0.16,^61^ 1.24±0.15,^4^ and 1.72 *μ*m.^62^ Typically, there is a thinner side and a thicker side, but we will use a symmetric model where both sides are equal. We choose d=0.4 μm, which makes $D_{0}=d/b=0.133$ and, consequently, $\lambda =L/d=1/\varepsilon D_{0}=1250$.

We treat the capillary blood as a uniform Newtonian fluid of apparent viscosity, μ, which takes into account the presence of red blood cells. This viscosity was modeled in macroscopic flows using elastic pellets for RBCs, apparently at room temperature, 22 °C.^63^ Using reasonable values of the hematocrit (0.45), the ratio of red blood cell diameter to channel depth (0.79), and plasma viscosity of 0.013 poise, their resulting curve-fit equation yields μ=0.025  poise. To account for the reduction of viscosity with increasing temperature, we settle on μ=0.02  poise. Interstitial fluid viscosity of rabbits was found to be $\mu_{i}=0.013\;\;\text{poise}$ at 37 °C,^64^ not surprisingly similar to the viscosity of lymphatic fluid $\mu_{\text{lymph}}=0.012\;\;\text{poise}$ in dogs.^65^ Consequently, the dimensionless viscosity ratio parameter is $\gamma =\mu_{i}/\mu =0.65$.

Physiologists use the Starling equation at the capillary membrane, $v=k_{c}\left(p-p_{i}-\sigma_{c}(\pi -\pi_{i})\right)$, to interpret and predict pulmonary fluid balance including edema. To do so, they assume p and $p_{i}$ are constants. While a constant (average) pulmonary capillary blood pressure can be reasonably estimated from pulmonary catheter wedge pressures, measuring $p_{i}$ is a technical challenge due to the small dimensions of the alveolar interstitium. Micropipettes of 2−3 μm diameter tips have been inserted into the interstitium surrounding >30 μm diameter blood vessels, a region known as the periadvential space with a representative value of −7.35  mmHg.^66,67^ Since our model has spatially varying $p_{i}$, this value is assigned to the end boundary pressures, $p_{i}(x=0)=p_{i}(x=L)=p_{iB}=-7.35\;\;\mathrm{mmHg}$. For capillary pressures, an average value of 6.6 mm Hg has been measured.^68^ Within this range, we choose $p_{a}=9,\;p_{v}=6\;\mathrm{mmHg}$, which makes $P_{a}=p_{a}/\left(p_{a}-p_{v}\right)=3$, $P_{v}=p_{v}/\left(p_{a}-p_{v}\right)=2$, and $P_{iB}=p_{iB}/\left(p_{a}-p_{v}\right)=-2.45$. For our channel flow, the average velocity is calculated as $u_{\text{avg}}=\left(p_{a}-p_{v}\right)b^{2}/\left(3\;\mu l\right)=0.12\;cm/s$, which is similar to measurements $u_{\text{avg}}=0.075\;cm/s$ for surface alveolar capillaries of rabbit lungs.^69^

Capillary hydraulic conductivity has been measured as $k_{c}=1\times \;10^{-7}cm/\left(\mathrm{mmHg}\;s\right)$ in frog muscle,^70^ $2-20\;x\;10^{-7}cm/\left(\mathrm{mmHg}\;s\right)$ in frog mesentery,^71^ $1.7\times 10^{-7}cm/\left(\mathrm{mmHg}\;s\right)$ in rat mesentery,^72^ and $0.36\;\times \;10^{-7}/\left(\mathrm{mmHg}\;s\right)$ in rat hindquarter.^73^ The last value was derived^74^ from the original data.^73^ From this range, we select a base value of $k_{c}=1\times 10^{-6}cm/\left(\mathrm{mmHg}\;s\right)$ and note that $1\;\;\mathrm{mmHg}=1333\;\;\text{dyn}/cm^{2}$ for adjusting the units.

Measurements of alveolar epithelial hydraulic conductivity in bullfrog lungs include $k_{A}=2.85\left(\pm 0.84\right)\;\;x\;10^{-7}$^75^ and $3.51\;\left(\pm 0.44\right)\times \;l0^{-8}-10^{-7}\;cm/\left(\mathrm{mmHg}\;s\right)$^76^ The bullfrog lungs are hollow sacs with no airway tree or alveolar structure, making the epithelial transport surface area estimates much simpler. From this range, we choose a base value of $k_{A}=5\times \;10^{-8}cm/\left(\mathrm{mmHg}\;s\right)$, which is 1/20th the value of k_c_. These choices yield $\kappa_{c}={\left(3k_{c}\mu D^{2}/1333\;b\right)}^{1/2}=5.16\times 10^{-5}$.

Alveolar septal interstitial permeability, K, is not reported directly in the literature. In general, K varies across different tissues over 3–4 orders of magnitude and depends on a number of factors including the state of hydration, pressure, stretch, and presence of disease. In rat abdominal muscle, $K/\mu =15-78\times 10^{-8}cm^{2}/\left(\mathrm{mmHg}\;s\right)$,^77^ which computes to $K=1.46-7.6\times 10^{-12}cm^{2}$. For rabbit aorta and intima, the results are $K=2.53\times 10^{-14}\;cm^{2}$.^78^ Rat subcutaneous tissue has $K=4.35\times 10^{-14}\;cm^{2}$,^79^ while dog subcutaneous interstitium has $K=2.3\times 10^{-11}\;cm^{2}$^80^ as interpreted.^81^ From this range, we choose $K=1\times 10^{-13}cm^{2}$, which makes the Darcy number $Da=K/d^{2}=6.25\times 10^{-5}$. Now we can compute the values $\kappa_{A}={\left(k_{A}\mu_{i}d/1333\;K\right)}^{1/2}=0.014$ and $\beta =\varepsilon^{2}\gamma /Da\;D=2.81$.

There are consistent data measuring, $p_{AL}$, while varying transpulmonary pressure, $\mathrm{TPP}=p_{AG}-p_{PL}$, by inflation where $p_{PL}$ is the pleural surface pressure. This was done in isolated lungs of adult rabbits,^82^ and mature and immature fetal rabbits^83^ and dogs.^84^ For isolated lungs, the pleural surface is surrounded by atmospheric air, so $p_{PL}=0$. In other studies, five values of $\mathrm{TPP}=5,\;10,\;15,\;20,\;\text{and}25\;cmH_{2}O$ yielded $p_{AG}-p_{AL}=1.8,6.2,9.5,13.3,16.8\;\;cmH_{2}O$, respectively, in the 31-day mature fetal rabbits.^83^ We can consider $\mathrm{TPP}=5\;\;cmH_{2}O$ as corresponding to an intact normal lung at end expiration. However, in that case, $p_{PL}=-5\;\;cmH_{2}O$ and $p_{AG}=0$. Therefore, $p_{AL}=-1.8,-6.2,-9.5,-13.3,-16.8\;\;cmH_{2}O$, noting that the $p_{AL}<0$ and increasingly negative for larger lung volumes. The jump in pressure across the air–liquid interface is due to the surface tension, σ, roughly as Laplace's law $p_{AL}=p_{AG}-2\sigma /R$, where R is the alveolar radius to the interface, see Fig. 2. In surfactant deficiency, as occurs in premature birth or COVID-19, the surface tension σ increases. In acute respiratory distress syndrome (ARDS), the present surfactant can be made ineffective due to the inflammation. Additionally, σ varies with lung volume^85–87^ as the interfacial surfactant concentration reduces with increasing surface area. That results in higher σ as the lung inflates. A normal value at FRC is found in Ref. 85 to be σ=4 dyn/cm for cat, dog, rabbit, and rat, by contrast Ref. 87 finds σ<1 dyn/cm at FRC in rabbit lungs. At this stage, we will simply use the measured $p_{AL}=-1.47\;\mathrm{mmHg}$ at FRC, so that $P_{AL}=p_{AL}/\left(p_{a}-p_{v}\right)=-0.49$.

The value of pulmonary interstitial osmotic pressure in rabbits was found to be $\pi_{i}=10.15\;\;\mathrm{mmHg}$,^67,88,89^ which we will assign. In the same study, capillary osmotic pressure was π=24.8  mmHg,^88^ while in humans, the data were π=25.4  mmHg.^90^ From these two values, we set π=25 mmHg. The lung liquid is generally void of large molecules, so we will assume $\pi_{AL}=0$. Reasonable values of the capillary and alveolar reflection coefficients are $\sigma_{c}=\sigma_{A}=0.80$.^91,92^ From this information, we compute $S_{A}=\sigma_{A}\left(\pi_{i}-\pi_{AL}\right)/\left(p_{a}-p_{v}\right)=2.71$ and $S_{c}=\sigma_{c}(\pi -\pi_{i})/\left(p_{a}-p_{v}\right)=3.96$. Finally, assuming a blood density of 1.06 g/cm^3^, the capillary Reynolds number is $\text{Re}=\rho bU_{s}/\mu =0.0057$, so we can ignore its effects, as was assumed in Eq. (8).

There are 13 independent dimensionless parameters based on 16 dimensional parameters, and there are two given dimensionless parameters, $\sigma_{c}=0.8\;\text{and}\;\sigma_{A}=0.8$. Other dimensional parameters that arise, which are not independent, are the pressure scale, $P_{s}/\varepsilon =\mu U_{s}/\varepsilon b={(p}_{a}{-p}_{v})$, the capillary velocity scale, $U_{s}{=b}^{2}{(p}_{a}{-p}_{v})/\mu L$, and the interstitial fluid velocity scale $W_{s}{=K(p}_{a}{-p}_{v})/\mu_{i}d$. Other dimensionless parameters that arise, which are not independent, are the aspect ratio, λ=L/d=1/εD=1250, the Darcy number, $Da=K/d^{2}=6.25\times 10^{-5}$, and the eigenvalues $k_{n}=n\pi /\lambda$. See Nomenclature for definitions of the dimensional and dimensionless parameters, respectively, and their base values. See Nomenclature for definitions of the dimensional and dimensionless variables.

## Data Availability

The data that support the findings of this study are available within the article.

## NOMENCLATURE

b: Capillary height (2b), 3 *μ*m
d: Interstitium thickness (4d), 0.4 *μ*m
D = d/b: 0.1333
Da = K/d^2^: 6.25 × 10^–5^
k_A_: Alveolar membrane hydraulic conductivity, 5 × 10^–8 ^cm·mmHg^−1^·s^−1^
k_c_: Alveolar membrane hydraulic conductivity, 1 × 10^–6 ^cm·mmHg^-1^·s^-1^
k_n_ = nπ/λ: wave number component n
K: Interstitial permeability, 10^–13^ cm^2^
L: Capillary length, 500 *μ*m
p: Capillary blood pressure
p_a_: Arterial pressure, 9 mmHg
p_AL_: Alveolar liquid pressure, −1.47 mmHg
p_i_: Interstitial fluid pressure
p_iB_: Interstitial fluid end pressure, −7.35 mmHg
p_v_: Venous pressure, 6 mmHg
P_s_/ε: *μ*U_s_/εb = (p_a_-p_v_) Pressure scale, 3 mmHg
P: p/(p_a_-p_v_) Capillary blood pressure
P_a_: p_a_/(p_a_-p_v_) 3
P_AL_: p_AL_/(p_a_-p_v_) −0.49
P_iB_: p_iB_/(p_a_-p_v_) −2.45
P_i _: p_i_/(p_a_-p_v_) Interstitial fluid pressure
P_v_: p_v_/(p_a_-p_v_) 2
Re: ρbU_s_/*μ* 0.0057
S**_A_**: σ_A_(π_i_-π_AL_)/(p_a_-p_v_) 2.71
S**_c_**: σ_c_(π-π_i_)/(p_a_-p_v_) 3.96
u: Capillary blood X-velocity
u_I_: Interstitial fluid X-velocity
U = u/U_s_: Capillary blood X-velocity
U_s_ = b^2^(p_a_-p_v_)/μl: Capillary velocity scale, 0.36 cm/s
U_i_ = u_i_/W_s_: Interstitial fluid X-velocity
v: Capillary blood Y-velocity
v_I_: Interstitial fluid Y′-velocity
V_i_ = v_i_/W_s_: Interstitial fluid η-velocity
V = v/εU_s_: Capillary blood Y-velocity
W_s_ = K(p_a_-p_v_)/μ_i_d: Interstitial fluid velocity scale, 7.7 *μ*m/s
x: Horizontal coordinate
X = x/L: Capillary horizontal coordinate
y: Capillary vertical coordinate
y′: Interstitial vertical coordinate
Y = y/b: Capillary vertical coordinate
β = ε^2^γdb/K: 2.81
γ = μ_i_/μ: 0.65
ε = b/L: 0.006
η = y′/d: Interstitial vertical coordinate
κ_A_ = (k_A_μ_i_ d/K)^1/2^: 0.014
κ_c_ = (3k_C_μD^2^/b)^1/2^: 0.000 051 6
λ = L/d = 1/εD: 1250
*μ*: Blood viscosity, 0.02 poise
*μ*_i_: Interstitial fluid viscosity, 0.013 poise
ξ: x/d Interstitial horizontal coordinate
π: Blood osmotic pressure, 25 mmHg
π_AL_: Alveolar liquid osmotic pressure, 0 mmHg
π_i_: Interstitial fluid osmotic pressure, 10.15 mmHg
ρ: Blood density, 1.06 g/cm^3^
σ_A_: 0.8
σ_c_: 0.8
